# Influence of Pragmatic Designs on Estimation of Clinical Effects among Randomized Trials in Aging

**DOI:** 10.1093/geroni/igaf122.2041

**Published:** 2025-12-31

**Authors:** Dana Weisenfeld, Thomas Travison

**Affiliations:** Hebrew SeniorLife, Boston, Massachusetts, United States; Hebrew SeniorLife & Harvard Medical School, Hebrew SeniorLife, Massachusetts, United States

## Abstract

There is broad acceptance that an intervention’s ‘real-world’ effectiveness is attenuated relative to its efficacy, but few articulations of what degree of reduction may be expected. We sought to determine the proportionate decrease in effects measuring effectiveness relative to efficacy, as well as the degree to which pragmatic trials may be underpowered in estimating clinically relevant effects in at-risk populations. To do so, we conducted a systematic search identifying 200 two-arm pragmatic trials, as well as 171 explanatory trials, published in high-impact biomedical journals from 2010-2024. Of these, 57 (29%) concerned aging populations and/or aging-related illnesses; twenty-eight (14%) described pharmacologic agents, while the remainder dealt with non-pharmacologic interventions. For each trial, the hypothesized ‘planning’ effect informing its power computation was isolated and the standardized treatment effect it estimated obtained. Of the pragmatic trials, relatively few were preceded by a companion efficacy trial. A total of 83 (42%) pragmatic trials demonstrated statistically significant evidence in favor of an intervention’s effect relative to control, while the corresponding proportion was 94 (55%) among the efficacy trials. Among the efficacy trials, the median ratio of the estimated effect to the planning effect was 0.60 (interquartile interval 0.07 to 1.07), while in the pragmatic trials this ratio was diminished, at 0.44 (interquartile interval 0.06 to 0.89). This review provides a first estimation of the impact of pragmatism on measures of effectiveness relative to efficacy in contemporary pragmatic trials in aging. Implications for the design of pragmatic trials in aging populations will be discussed.

